# Perforated Duodenal Ulcer in the Immediate Postpartum Period: Diagnostic Pitfalls Following Caesarean Delivery

**DOI:** 10.7759/cureus.106462

**Published:** 2026-04-05

**Authors:** Jordan De Guzman, Jacob Mar, Michael Jacobs

**Affiliations:** 1 Department of Surgery, Henry Ford Providence and Novi Hospitals, American University of the Caribbean, Cupecoy, SXM; 2 Department of Surgery, Henry Ford Providence Southfield and Novi Hospitals, Southfield, USA

**Keywords:** cesarean ​section, diagnostic dilema, duodenal ulcers, ruptured peptic ulcer, unexplained abdominal pain

## Abstract

Postpartum abdominal pain is frequently encountered and is most often related to obstetric causes such as uterine involution, infection, or postoperative discomfort following cesarean delivery; however, more atypical etiologies can also occur. Perforated peptic ulcer disease in the immediate postpartum period is uncommon and may be overlooked, particularly when symptoms overlap with expected postoperative findings and recent surgical history. We report a woman in her early 30s who developed severe upper abdominal pain four days after a primary cesarean delivery performed for maternal exhaustion in the setting of intraamniotic infection. Her initial postoperative course was uncomplicated, and she was discharged on scheduled ibuprofen and acetaminophen for pain control. On presentation, she was tachycardic with elevated liver enzymes, raising concerns for hemolysis, elevated liver enzymes, and low platelets (HELLP) syndrome, biliary pathology, or postoperative infection. MRI and CT imaging demonstrated pneumoperitoneum and ascites, findings that were initially attributed to recent surgery. Due to persistent symptoms, a diagnostic laparoscopy was performed and revealed a 1.5 cm perforated duodenal ulcer with diffuse peritonitis. The ulcer was repaired with oversewing and a Graham patch, and the patient recovered following appropriate surgical and medical management; Helicobacter pylori testing prior to discharge was negative. This case highlights that a perforated peptic ulcer should remain in the differential diagnosis for postpartum patients presenting with persistent abdominal pain, even in the absence of classic risk factors, as recent cesarean delivery can complicate imaging interpretation and delay diagnosis, underscoring the importance of early multidisciplinary evaluation and prompt surgical intervention to reduce morbidity.

## Introduction

Postpartum abdominal pain is common and most frequently results from obstetric and postoperative causes such as uterine involution, endometritis, and incisional pain following cesarean delivery. As the most common abdominal surgery performed worldwide, cesarean delivery is associated with postoperative discomfort, infection, and transient imaging abnormalities, all of which may contribute to diagnostic ambiguity in the early postpartum period [1]. As a result, non-obstetric causes of abdominal pain may be underrecognized, including conditions such as acute appendicitis, cholecystitis, and pancreatitis, as well as less common but potentially life-threatening entities such as perforated peptic ulcer disease [2].

Complicated peptic ulcer disease with anterior duodenal perforation is rare in the postpartum setting, with limited data describing its incidence. Its presentation can be nonspecific and may overlap with more common obstetric and postoperative conditions, contributing to diagnostic delay.

We present a case of a postpartum patient who developed persistent abdominal pain following cesarean delivery, initially raising concern for HELLP syndrome, hepatobiliary pathology, and pelvic abscess, but who was ultimately found to have a perforated duodenal ulcer requiring surgical intervention. This case highlights the diagnostic challenges and potential pitfalls in evaluating postpartum abdominal pain, serving as a reminder that diagnostic vigilance must extend beyond obstetric considerations, as postoperative changes may obscure underlying non-obstetric pathology.

## Case presentation

A 31-year-old G1P0 woman at 39.5 weeks’ gestation with no history of tobacco use or peptic ulcer disease underwent induction of labor for insulin-controlled gestational diabetes mellitus. Her pregnancy was additionally complicated by gestational thrombocytopenia and polyhydramnios. During labor, she developed intraamniotic infection and was treated with intravenous ampicillin and gentamicin. After three hours of pushing with minimal fetal descent, she underwent a primary low-transverse cesarean section for maternal exhaustion. The procedure was completed without intraoperative complications.

She was discharged with a postoperative pain regimen that included 650 mg of acetaminophen, 5 mg oral tablets of oxycodone, and scheduled ibuprofen 600 mg every 6 hours. She had been taking ibuprofen at a total daily dose of 2400 mg for approximately four days prior to symptom onset. Clindamycin was added to treat the intraamniotic infection. On postoperative days one and two, she remained clinically stable, ambulating, tolerating her diet, and reporting well-controlled pain.

On postoperative day four, she developed new-onset severe upper abdominal pain rated 8/10 in intensity. The pain was sharp, radiated to the right shoulder, and was exacerbated by eating and deep inspiration. Associated symptoms included chills, nausea, and indigestion. After inadequate relief with oral oxycodone, she presented to the emergency department.

On arrival, vital signs were notable for a heart rate of 110 beats per minute. Laboratory evaluation, summarized in Table 1, demonstrated mild transaminitis without leukocytosis or thrombocytopenia. Given her postpartum status, epigastric pain, and abnormal liver enzymes, obstetric etiologies such as HELLP syndrome were considered, along with postpartum infection, biliary pathology, and postoperative complications. Initial abdominal ultrasound did not demonstrate free intraperitoneal fluid or other acute findings, further contributing to diagnostic uncertainty. On hospital day five, she developed a fever of 101.1°F and a heart rate of 116 beats per minute. Physical examination revealed abdominal distension with tenderness in the upper abdomen and periumbilical region, accompanied by rebound tenderness. Serial laboratory evaluation demonstrated fluctuating transaminase levels without progression to severe hepatocellular injury or accompanying hyperbilirubinemia or leukocytosis, further complicating diagnostic interpretation.

**Table 1:** Laboratory findings on postoperative day four following cesarean delivery. Laboratory values are shown with corresponding institution-provided reference ranges. Findings demonstrate mild transaminitis without leukocytosis or thrombocytopenia. WBC: White Blood Cells, AST: Aspartate Aminotransferase, ALT: Alanine Aminotransferase, LDH: Lactate Dehydrogenase

Test	Value	Units	Reference Range
WBC	10.2	×10³/µL	4.00–11.00 ×10³/µL
Hemoglobin	9.6	g/dL	12.0–16.0 g/dL
Platelets	206	×10³/µL	150–400 ×10³/µL
Sodium	138	mmol/L	136–145 mmol/L
Creatinine	0.6	mg/dL	0.5–1.0 mg/dL
Total bilirubin	0.3	mg/dL	0.1–1.2 mg/dL
Alkaline phosphatase	125	U/L	20–120 U/L
AST	71	U/L	10–35 U/L
ALT	117	U/L	10–35 U/L
Albumin	3.3	g/dL	3.5–5.2 g/dL
LDH	252	U/L	135–225 U/L

**Figure 1:**
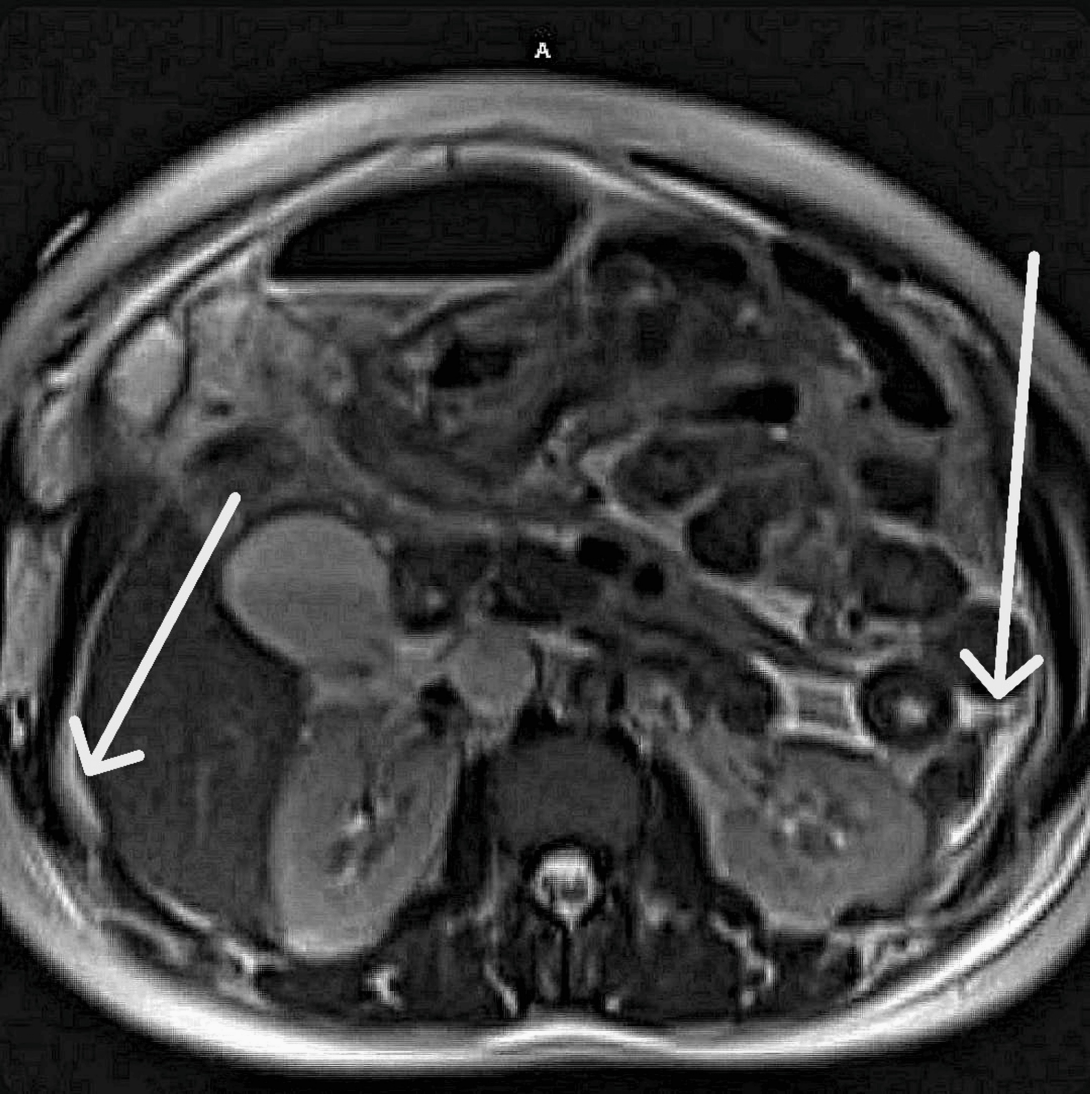
Axial T2-weighted MRI of the abdomen demonstrating moderate ascites. Axial T2-weighted MRI of the abdomen demonstrating moderate-volume ascites. The left arrow indicates perihepatic free fluid, while the right arrow highlights additional intraperitoneal fluid within the abdomen. Gaseous distension of bowel loops is also noted.

**Figure 2:**
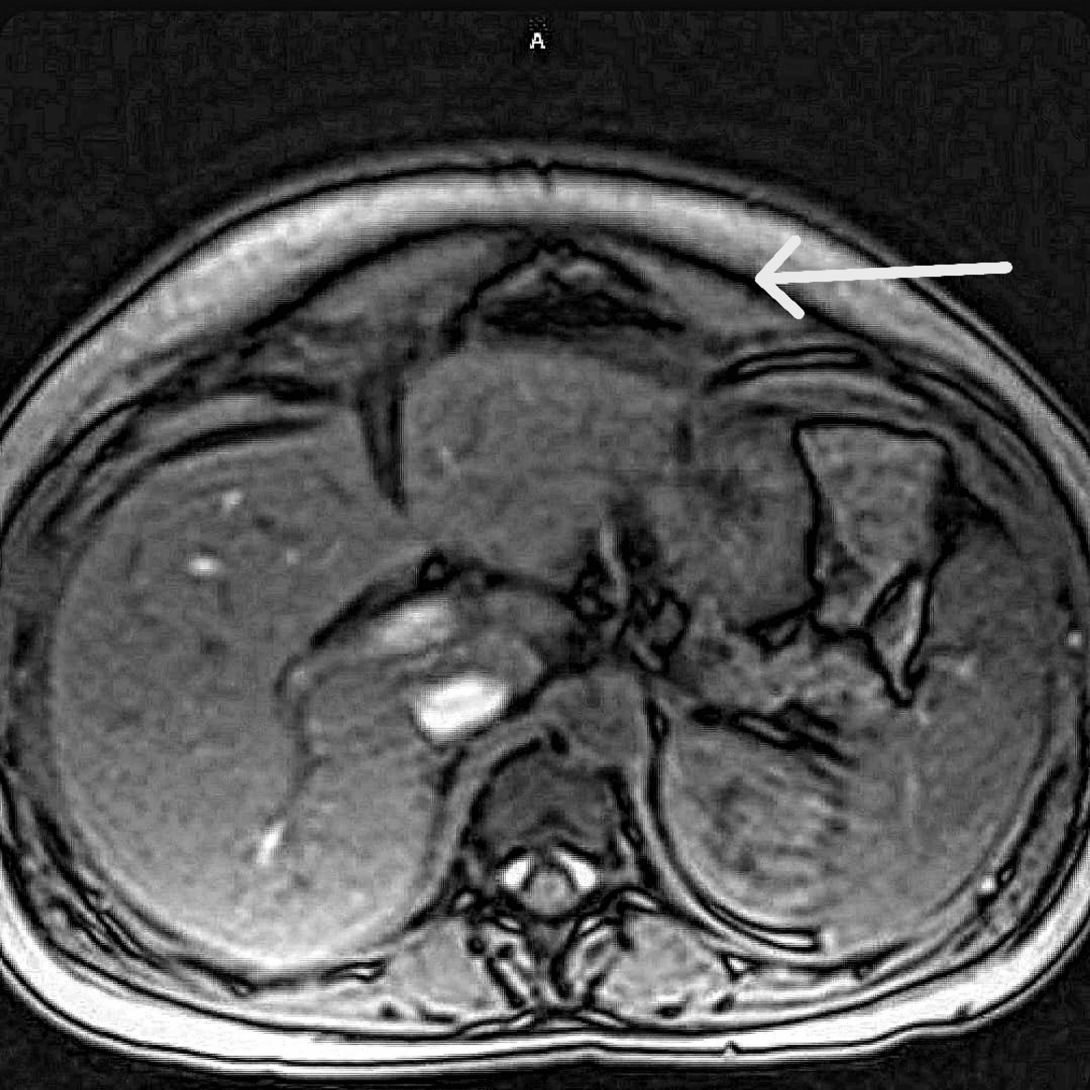
Axial T1-weighted MRI of the abdomen demonstrating possible free air at the anterior abdominal wall. Axial T1-weighted MRI of the abdomen with an arrow pointing at a focus of signal void, suggesting moderate volume intraperitoneal air in the upper central abdomen. Given the recent cesarean delivery, these findings were initially considered potentially postoperative in nature, contributing to diagnostic uncertainty.

Obstetric consultation ruled out HELLP syndrome and found no clinical evidence of endometritis. Gastroenterology evaluation noted epigastric pain worsened by food intake and mild transaminitis without clear evidence of biliary pathologies such as bile duct dilation, cholelithiasis, or choledocholithiasis. MRI of the abdomen demonstrated moderate ascites and pneumoperitoneum (Figures 1, 2). Although pneumoperitoneum can be observed following cesarean delivery, these findings prompted further evaluation. 

**Figure 3:**
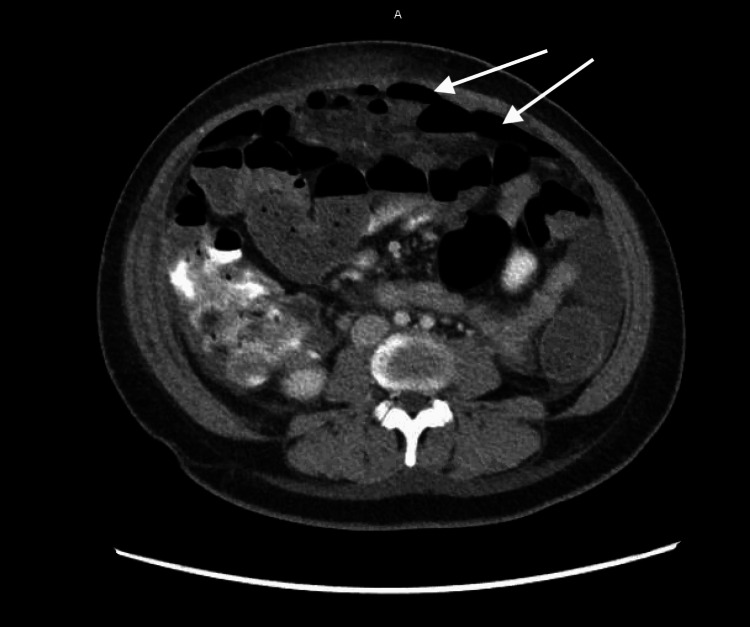
Contrast-enhanced CT of the mid-abdomen (axial). Arrows point to free intraperitoneal air consistent with pneumoperitoneum, most prominent in the anterior abdomen, concerning for a perforated viscus.

**Figure 4:**
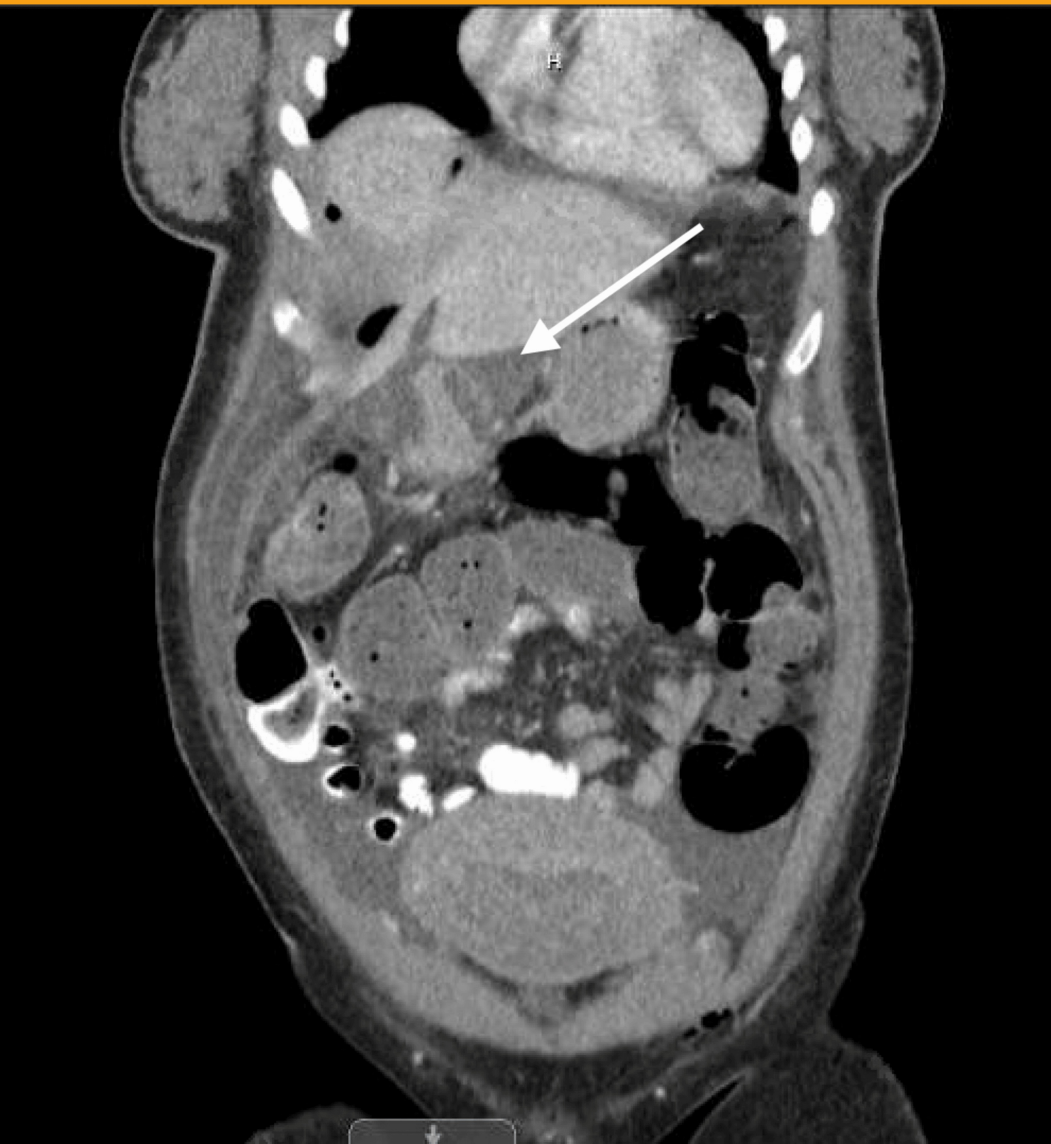
Contrast-enhanced CT abdomen and pelvis (coronal view). The white arrow indicates a fluid-containing structure along the anterior abdominal wall, which may represent bowel injury versus a developing postoperative fluid collection or abscess.

Subsequent CT imaging of the abdomen and pelvis revealed free intraperitoneal air, ascites, and a 4.5 × 2.9 cm fluid-containing structure along the anterior abdominal wall, interpreted as a possible bowel versus a developing postoperative fluid collection or abscess (Figures 3, 4). These findings raised concern for an intra-abdominal infectious process without clearly identifying a perforated viscus.

**Figure 5:**
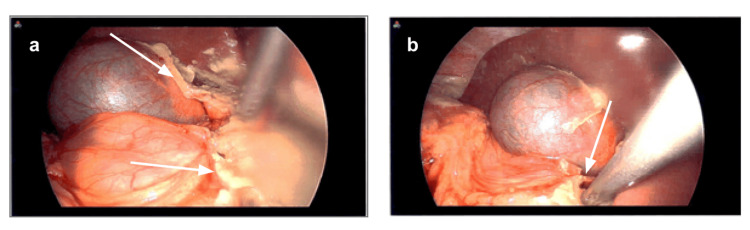
Intraoperative laparoscopic findings demonstrating duodenal perforation and associated peritonitis. Intraoperative laparoscopic images demonstrating (a) diffuse inflammatory changes with surrounding fibrinous exudate (arrow) and (b) the perforation site indicated by the instrument tip (arrow). Findings are consistent with perforated viscus and diffuse peritonitis.

Given persistent clinical concern, the patient underwent diagnostic laparoscopy. Intraoperatively, diffuse fibrinous exudative peritonitis with extensive inflammatory changes was observed (Figure 5a). The source of contamination was identified as a 1.5 cm anterior perforation at the junction of the first and second portions of the duodenum (Figure 5b). The abdomen was irrigated, the ulcer was oversewn, and the defect was repaired using a Graham patch with a falciform flap. A drain was placed through the left upper quadrant of the abdomen at the dissection bed, and the patient was transferred to the intensive care unit for postoperative monitoring.

In the ICU, she was treated with a proton pump inhibitor and empiric broad-spectrum antibiotics and demonstrated progressive clinical improvement. By postoperative day two, she was ambulating, voiding independently, and tolerating the return of bowel function. 

On postoperative day four, she underwent an upper gastrointestinal contrast study with gastrograffin to assess for extravasation at the repair site. The findings were negative for extravasation, and oral intake was subsequently advanced. Prior to discharge, *Helicobacter pylori* testing came back negative. Six days after diagnostic laparoscopy, she was discharged on a full liquid diet with close outpatient follow-up.

## Discussion

Pregnancy and the postpartum period are characterized by substantial physiologic and hormonal changes that can alter symptom interpretation and complicate clinical assessment. Abdominal pain in the postpartum period is common and most frequently benign [3]. It is often attributed to obstetric causes such as uterine cramping and involution, perineal lacerations, and incisional pain following cesarean delivery [4,5]. However, a subset of women experience persistent or progressive abdominal pain that may signal serious underlying pathology. Obstetric conditions, including uterine rupture, puerperal infection, abscess formation, postpartum hemorrhage, HELLP syndrome, and eclampsia, often dominate early diagnostic evaluation because of their temporal association with parturition and high prevalence in the immediate postpartum period [4,5]. The nonspecific nature of postpartum abdominal pain, combined with this clinical focus on obstetric etiologies, creates a diagnostic environment in which potentially life-threatening non-obstetric conditions may be underrecognized [6-9]. Accordingly, recognition of both obstetric and non-obstetric causes of postpartum abdominal pain is essential to prevent diagnostic delay and adverse outcomes.

Among the less common non-obstetric causes of acute postpartum abdominal pain is perforated peptic ulcer disease. Although peptic ulcer disease (PUD) carries an estimated lifetime risk of approximately 5% to 10% in the general population, perforation during pregnancy and the immediate postpartum period remains rare, with limited contemporary literature describing such cases [7-9]. Reports specifically describing perforated ulcers following cesarean delivery remain limited [10,11]. For example, Levin et al. reported a post-cesarean duodenal perforation case, highlighting that, although uncommon, this condition warrants consideration in the differential diagnosis of acute postpartum abdominal pain [3].

Established risk factors for PUD include *Helicobacter pylori* infection, chronic or high-dose nonsteroidal anti-inflammatory drug (NSAID) use, advanced age, prior ulcer disease, and smoking [10,12]. Among these, H. pylori infection and NSAID exposure are most consistently implicated in duodenal ulcer formation. However, the relative contributions of these factors during the peripartum period remain incompletely characterized. In the absence of classic risk profiles, the development of perforated PUD in young postpartum patients raises consideration of multifactorial contributors, including physiological stress, infection, and transient alterations in mucosal protection.

This case illustrates a perforated duodenal ulcer in a young postpartum patient without a longstanding history of peptic ulcer disease or other major predisposing factors, aside from short-term postoperative NSAID therapy. The patient experienced several peripartum stressors, including prolonged labor, intraamniotic infection, and cesarean delivery, which may have increased physiologic stress and contributed to ulcer formation. Acute systemic inflammation and perioperative stress are associated with impaired gastric mucosal defense and increased susceptibility to ulcer formation. In addition to inflammatory contributors, hormonal influences may also be relevant [7,8,11,13,14]. Pregnancy is considered a relatively protective state against peptic ulcer disease, likely due to hormonal effects on mucosal integrity; however, the persistence of these protective mechanisms into the immediate postpartum period remains incompletely defined [9,11]. These factors demonstrate how multifactorial stressors in the peripartum period may increase vulnerability to uncommon intra-abdominal pathology while simultaneously obscuring its recognition within a clinical framework focused on obstetric considerations.

In this case, diagnostic complexity arose due to significant overlap with more common postpartum etiologies, complicating early identification of the underlying pathology. Initial concerns included HELLP syndrome, abscess formation secondary to chorioamnionitis, and hepatobiliary disease, all of which can present with acute abdominal pain after delivery [6,8,9,12,13]. On presentation, the patient was tachycardic and reported chills, nausea, and indigestion. Upper abdominal pain with mild transaminitis further increased concern for HELLP syndrome or choledocholithiasis. Obstetric and gastroenterology consultations excluded these conditions, as there was no evidence of hyperbilirubinemia, thrombocytopenia, or other features of primary hepatobiliary disease; the elevated liver enzymes were instead attributed to a reactive process in the setting of intra-abdominal inflammation.

In the absence of a definitive explanation, imaging was pursued. MRI followed by CT demonstrated pneumoperitoneum and ascites, findings considered possibly postoperative due to the recent cesarean delivery. Additionally, a 4.5 × 2.9 cm fluid-containing structure along the inner anterior abdominal wall was identified, raising concern for a bowel loop or a developing postoperative fluid collection or abscess. Despite serial examinations, laboratory evaluation, and cross-sectional imaging, a clear etiology for the persistent abdominal pain remained elusive. Diagnostic laparoscopy was therefore performed, ultimately revealing a perforated duodenal ulcer and enabling definitive surgical management.

This case accentuates the importance of maintaining a broad differential diagnosis when evaluating postpartum abdominal pain. Although obstetric etiologies predominate during this period, atypical non-obstetric causes such as perforated peptic ulcers should be considered, particularly in the context of surgical stress, intra-amniotic infection, and NSAID exposure. In this patient, interpretation of imaging findings was complicated by recent cesarean delivery, as postoperative pneumoperitoneum and fluid collections were initially attributed to expected surgical changes. Early consultation with surgical and obstetric specialists is essential, as delays in recognizing perforated ulcers are associated with significant morbidity. As noted by Shirazi et al., new-onset tachycardia accompanied by abdominal distension following cesarean delivery should prompt concern for severe intra-abdominal pathology, including peptic ulcer perforation. Increased provider awareness of uncommon disease processes in the postpartum period may reduce diagnostic delay and improve patient outcomes.

## Conclusions

Perforated peptic ulcer disease is a rare but important consideration in postpartum patients presenting with persistent abdominal pain after cesarean delivery. This case illustrates the diagnostic challenges in this population, where symptoms often overlap with common obstetric and postoperative conditions, making less frequent but serious pathologies harder to recognize. In particular, postoperative pneumoperitoneum, nonspecific imaging findings, and competing diagnoses such as HELLP syndrome, hepatobiliary disease, or intra-abdominal infection contributed to initial diagnostic uncertainty. It also emphasizes the need to maintain a broad differential diagnosis and to reassess the working diagnosis when a patient’s clinical course does not improve as expected, with early surgical evaluation when appropriate. Early recognition of atypical causes of postpartum abdominal pain is essential to avoiding delays in definitive management and reducing morbidity.
